# Patient and care partner views on exercise and structured physical activity for people with Progressive Supranuclear Palsy

**DOI:** 10.1371/journal.pone.0234265

**Published:** 2020-06-05

**Authors:** Susan C. Slade, Christopher Bruce, Jennifer L. McGinley, Bastiaan R. Bloem, Meg E. Morris

**Affiliations:** 1 La Trobe Centre for Sport and Exercise Medicine Research, School of Allied Health, Human Services & Sport, SHE College, La Trobe University, Bundoora, Australia; 2 Discipline of Occupational Therapy, School of Allied Health, Human Services & Sport, SHE College, La Trobe University, Bundoora, Australia; 3 Physiotherapy, The University of Melbourne, Parkville, Australia; 4 Department of Neurology, Radboudumc Center of Expertise for Parkinson & Movement Disorders, Donders Institute for Brain, Cognition and Behaviour, Radboud University Medical Center, Nijmegen, the Netherlands; 5 Healthscope, Victorian Rehabilitation Centre Healthscope, Glen Waverley, Australia; Philadelphia VA Medical Center, UNITED STATES

## Abstract

**Introduction:**

Progressive Supranuclear Palsy (PSP) is a debilitating form of atypical Parkinsonism. People living with PSP experience movement disorders affecting walking, balance and eye movements. The role of exercise in optimising movement remains unclear.

**Aims:**

To identify beliefs about exercise and structured physical activity through the experience of people with PSP.

**Methods:**

Using a phenomenological theoretical framework, qualitative methods were employed to obtain the views of people living with PSP, and their care partners, by in-depth interviews. Questions derived from a systematic review and expert opinions guided the interviews which were audio-recorded, transcribed and de-identified. Two researchers independently conducted thematic analysis and reached consensus on emerging themes.

**Results:**

There were 16 participants. Four themes were identified: (i) there are beliefs and preferences about exercise and physical activity that can impact on engagement; (ii) difficulty coping with disease progression impacts activities; (iii) facilitators to exercise include individual preferences, access to facilities and expert advice; and (iv) perceived barriers include beliefs about limited exercise options, falls risk, cost, transport and access to credible information.

**Discussion:**

People living with PSP want early guidance about the condition and the role of exercise. It is important to quickly enable people to have access to evidence and resources supporting exercise and structured physical activities. Consideration of individual preferences and access to expert advice facilitate engagement. Individual barriers need to be identified and solutions found.

**Conclusion:**

People living with PSP are amenable to exercise, especially early in the disease process. Clear guidelines are warranted to document which exercises, and physical activities are most beneficial.

## Introduction

Progressive Supranuclear Palsy (PSP) is a debilitating and progressive form of atypical Parkinsonism (PD) [1–4]. People living with PSP typically experience movement disorders that affect walking, balance, eye movements and participation in social roles. However, the role of exercise in mediating disease progression and optimising movement remains unclear [3–6]. Cognitive impairment, falls, and depression are often associated with PSP progression, and may hamper participation in regular exercise and physical activity [7, 8]. People living with PSP often have co-morbidities such as osteoarthritis and cardiovascular disease, which can improve with exercise [9–11]. It is unclear whether an exercise intervention in the early to middle stages of PSP can influence health-related quality of life, optimise mobility or reduce caregiver strain.

There are several known challenges to implementing effective movement rehabilitation programs for people living with PSP. The diagnosis, and therefore therapies, can be delayed because ‘‘definite PSP” is only confirmed by post-mortem analysis, such as finding tau protein pathology in the brain [4, 5, 8]. Most people are designated as having “probable” or “possible” PSP [4, 5, 8]. This typically needs to be observed for a period of time, such as two to four years, before making the provisional diagnosis [2, 7]. It also remains unclear which forms of exercise, structured physical activities and movement rehabilitation are most beneficial for PSP given a paucity of research on this topic [12, 13].

A recent systematic review that evaluated exercise and physical activity for people with PSP showed emerging evidence of benefit from walking programs and balance training [12]. In the absence of clinical guidelines on exercise for PSP, clinicians sometimes deliver exercises known to be effective for people living with idiopathic PD. This is because some of the initial symptoms can appear to be similar [14–18]. Whether or not it is valid to use interventions for PD such as cueing [19–21], strength training [20, 22, 23], balance rehabilitation [24, 25], treadmill training [26, 27], aerobic exercises such as cycling [28] or even dancing [29, 30] for people living with PSP remains unclear.

Design of evidence-based interventions such as exercise programs and movement rehabilitation can be informed by a synthesis of the published literature from clinical trials as well as by consumer experiences, beliefs and preferences [31, 32]. Factors known to be associated with exercise behaviour in PD include movement disorders [27, 33], self-efficacy [34], levels of motor or cognitive impairment [35, 36] and depression [34, 37]. Individual preferences are also important facilitators or barriers to exercise engagement for a range of health conditions. Consideration of consumer preferences is therefore a priority for developing programs that are tailored to the individual [38, 39]. There remains a need to identify facilitators and barriers to participation and engagement as well as optimal content, dosage and scheduling of exercises for people living with PSP [12].

The present study explores the potential benefits and perceived challenges of exercise through the experiences of people directly affected by this rare, chronic and progressive disease. We aim to identify beliefs about, and facilitators and barriers to, exercise and physical activity. We defined exercise as planned, structured, repetitive and intentional movement intended to improve or maintain strength and fitness [40]. We sought the views of people living with PSP, care partners and family members and developed the following research question: What is the lived experience of exercise and structured physical activity for people living with PSP?

## Materials and methods

### Theoretical framework

Understanding people’s beliefs about facilitators and barriers to exercise and physical activity is well suited to a qualitative method of enquiry [41–43]. In qualitative research, a phenomenological approach aims to explore personal lived experiences to help understand how people make sense of the world in which they live [44–46]. It provides a deep level of understanding that may not always be revealed through randomised controlled trials or responses in questionnaires and surveys [44–46]. This framework was applied in the current study to gain a deeper understanding of the perceptions of people diagnosed with PSP, and their caregivers and/or family members, about exercise and physical activity.

### Design overview

Individual interviews were conducted at home for people living with PSP, caregivers and family members. We did not conduct focus groups because of the burden of travelling to one location, communication difficulties amplified in a group setting and the sensitive nature of some personal disclosures. The study was reported according to the Consolidated Criteria for Reporting Qualitative studies (COREQ) checklist [47].

### Ethics considerations

The study was approved by the La Trobe University Human Research Ethics Committee (Project: HEC18333). All participants provided written informed consent prior to data collection. Participants were assured of confidentiality and anonymity and were assigned an identification number. All data within the transcribed recordings were de-identified for person and place. The participants were given the opportunity to speak freely and openly.

### Research team

Three members of the study team trained as physiotherapists and had a range of research and clinical expertise. SCS (PhD) had expertise in physiotherapy, exercise prescription and qualitative research methods. MEM (PhD) and JMG (PhD) had expertise in neurological physiotherapy, movement disorders, quantitative research, project management and supervision. CB (PhD) qualified as an occupational therapist and had expertise in neurological rehabilitation, evidence-based practice and data analysis. BRB (PhD) is an international expert neurologist specialising in movement disorders and Parkinson’s research. None of the research team members were known to any of the participants.

### Participant eligibility and recruitment

Recruitment of people with PSP, care partners and family members, was via purposive sampling in Australia and continued until data adequacy and saturation were reached. An advertisement and information summary were placed in the state-wide Parkinson’s association newsletter and distributed at atypical Parkinson’s support groups. Advertisements were also placed in the reception areas of movement disorders neurology and physiotherapy clinics. People living with PSP had to have received a provisional or final diagnosis of PSP from a medical practitioner and have verbal capability for audio-recording. There was no requirement of experience with exercise and there were no cognitive criteria, All patients, family members, and caregivers who contacted the researcher by email or phone were provided with an explanation of the study. The researcher answered any questions and provided potential participants with a consent form. The interviews followed completion of written informed consent and, for people living with PSP, provision of a signed doctor’s ethics approved screening form confirming a diagnosis of PSP and medical suitability to participate in an interview. Interviews were scheduled at a time convenient to each of the participants and were either in the home or via videoconferencing.

### Data collection

An experienced facilitator (SCS), unknown to any of the participants, conducted audio-recorded interviews. A set of interview questions was informed by a systematic review [12] and expert opinions from the research team as well as consulting with the Parkinson’s association representatives (Table 1). Members of the research team are international experts on movement disorders, PD and atypical parkinsonism, including PSP. There was no participant input or pilot testing due to the possibility of introducing bias in a very small potential participant pool. Questions guided conversation about the diagnostic experience, exercise preferences and practices (such as walking, aquatic, dance, strengthening), movement strategies and information needs. Demographic data such as age, time since diagnosis, medication, co-morbidities, falls incidence, exercise activity and beliefs/preferences and utilisation of allied health services were collected from participants. Care partners provided data such as age, exercise beliefs and practices, co-morbidities. Field notes were made immediately after each interview to provide a record of observations and impressions. These notes included non-verbal or emotional responses, observations and possible patterns emerging from the collected data. Data collection took place from January 2019 until December 2019.

**Table 1:** Interview guide.

1. What has been your experience about the diagnosis and information given about management options?
2. Do you know how, or where, to access information?
3. What do you believe/think about exercise, physical activity or therapy? (this may include prompts for past history/experience of exercise pre-disease onset, health conditions etc)
4. Do you participate in exercise, physical activity or therapy?
• If, yes—what type, how often, where, with whom
• Is this self-exercise or walking
• Is this physiotherapy or structured exercise or e.g. gym, exercise class, dancing, singing
• Do you use Smartphone apps or videos/DVDs
• If no, why not—e.g. access, fatigue
5. What would help you to participate in exercise, physical activity or therapy?
• Cost, scheduling, location, facilities, equipment
6. What exercise, physical activity or therapy would you like or not like to do?
7. Where would you prefer to exercise, be physically active or take part in therapy?
• Location/setting, access, equipment, supervisor/instructor
8. What concerns do you have about falling (or your balance)?
• Have you fallen or had “near misses”?
• How many in the past 12 months?
9. What exercise/activity advice/guidance have you had from health care providers?
• If yes—what?
• If no—what would you like, or need, to know?

### Data storage and confidentiality

Participants were de-identified by number and all hard copy data (master list, informed consent, demographic data and doctor’s screening forms) were stored securely in a locked filing cabinet in a locked office. Audio recordings and electronic de-identified verbatim transcriptions were stored on a secure university research drive.

### Data analysis

Two researchers (SCS and CB) independently conducted an iterative, step-by-step thematic analysis of the de-identified transcripts by using an inductive method [41, 43, 44, 48]. Transcripts were read several times to ensure familiarity. Data were coded and compared, categorized into clusters of meaning according to similarities. Each researcher independently developed preliminary themes by using descriptive content analysis and conducted documented video-conferencing and face-to-face meetings, followed by confirmation emails, until consensus was reached for the final set of themes and sub-themes. Representative quotations for each theme were selected and pooled together to support the identified themes. A coding-tree is available from SCS on request. The research team were available for consultation throughout the analysis process. MEM was consulted for content expertise and consensus.

### Bias control and method quality

Steps were taken to enhance the rigour of the study design [49]. These steps included: (1) *a priori* eligibility; (2) recruitment of participants who had the experiences to address the research questions and were also likely to bring different experiences based on stage of the condition; (3) documented and replicable data collection and data analysis steps; (4) audio-recording and verbatim transcription; (5) independent data analysis by two researchers and (5) results linked to participant data.

## Results

In-depth individual interviews were conducted with 16 participants (eight care partners and eight people living with a diagnosis of probable PSP; there were six couples, one mother-son, one widowed care-partner and one individual with PSP). Interviews were conducted by SCS either in the home or in the residential care facility. There were ideas and experiences common to all the interviewees and no dissonant reports. Repeat interviews and member checking of transcripts were not required as, for the former, data saturation was reached, and, for the latter, there was a risk of recall bias. There were no dropouts or refusals to participate.

The mean age of people living with PSP was 71.4 years (SD: 7.7) and of carers was 66.4 years (SD: 10.4) years. The mean time since diagnosis was 3.4 years (SD: 1.1) although cognitive deficits and falls existed for several years prior to provisional diagnosis in all people living with PSP. The mean interview time was 61.0 minutes. Two participants with PSP lived alone in residential care and six participants with PSP lived at home with a care partner/spouse. Falls were frequent and ranged from daily to once a month. Health comorbidities included type 2 diabetes, hypertension, cardiovascular disease, osteoarthritis, vision and speech impairments, sleep apnoea, Meniere’s disease, frontal lobe dementia, meningioma and back and neck pain (Table 2).

**Table 2:** Participant demographic data.

P	Interview time (mins)	Age	Sex	Role/Relationship	Dwelling	Time since diagnosis	Medication	Co-morbidities	Falls—last 12 months	Assistive devices	Exercise	HCP
11	66	78	F	PwPSP	R	3Y	Irbesartan, Prolia, Aspirin	HC, HT	7	Walker	Group ex– 1hr 3/week Unsupervised home ex—weights	GP, N, PT
											Ex—ambivalent	
12	66	46	M	CP/son	C	N/A	0	0	N/A	N/A	Gym	GP
											Ex—important	
13	44	65	F	PwPSP	C	5Y	Madopar, Metformin,	D (type 2), HT	12	0	Walking– 20mins, daily	GP, N, PT
											Ex—important	
14	44	62	M	CP/wife	C	N/A	N/A		N/A	N/A	Ex—important	GP
16	59.5	73		PwPSP	C	1Y 6 Y—CC	Candesartan Cartia, Nexterone, Symmetrel	D (type 2), HT, normal pressure hydrocephalus	20	None	Group ex (neuro)– 1hr, 1/week	DI, GP, N, OT, PT, SP
											Resistance ex (home PT)—1/week	
											Gym (unsupervised)– 1/week	
											Ex—important	
17	59.5	72	F	CP/wife	C	N/A	0	MD	N/A	N/A	Pilates– 2/week	GP, O
											Ex—important	
18	67	70	F	CP/PwPSP deceased	C	N/A	0	HT, OA	N/A	N/A	Walking—daily	GP
											Ex—important	
21	66.5	78	F	PwPSP/single	R	4Y	Enalapril, Ostelin, Propranolol, Stemetil, Thyroxin	DV, HT, MD, OA,	2 Daily—near misses	Walker, Motorised wheelchair, R wrist brace, R & L AFO	Group class– 3/week	GP, N, PT
											Unsupervised home ex Short walks	
											Ex—important but difficult (fatigue)	
23	59	58	M	CP/husband	C	N/A	0	0	N/A	N/A	Gym– 3/week Tandem bicycle– 3/week Ex—important (years of gym membership)	GP
24	59	56	F	PwPSP	C	4Y	Madopar, Buscopan	0	2–3 / week	Walker	Neuro PT– 1/2weeks	GP, N, OT, PT, SP
											Home ex (daily)–weights, stretching Tandem bicycle– 3/week	
											Ex—important (years of gym membership—not now)	
29	54.5	70	M	PwPSP	C	4Y	Symmetrel, Amantadine hydrochloride, Mirtazapine	Depression, OA	10	Walker	Gym– 3/week, Personal trainer– 1/wk,	GP, N, PT
											Walking—daily,	
											Ex is important	
30	54.5	71	F	CP/wife	C	N/A	0	0	0	N/A	Gym– 3/week,	GP
											Walking– 1km daily,	
											Ex is important	
31	69.5	73	F	PwPSP	C	6Y	Candesartan, Cartia, Sotalol, Moxonidine, Lercanidipine, Escitalopram, Esomeprazole, Temazepam	AF, CVD, G, HT, OA, HC, SA, Brainstem meningioma, depression	6 Ambulance called	Walker	Chair exercises– 3/week	CA, E, GP, N, O, OT, P, PT, SP
											Dancing—a past preference	
											Does not like ex	
32	69.5	76	M	CP/husband	C	N/A	0	0	0	N/A	Golf, walking, back exercises,	GP, PT
											Ex is important	
33	62	78	M	PwPSP	C	2Y 5 Y—CC	Crestor, Paxam, Duodart, Nizac, Propranolol, Ventolin	Asthma, OA, Frontal lobe dementia, R.THR, TSR (bilateral)	6 Ambulance called	Walker	Ex bicycle– 30 mins, 3/week,	GP, N, PT, SP
											Parkinson’s Movement class– 1/week, ParkinSong– 1/month, Unsupervised ex from PT,	
											Ex was important but now apathetic	
34	62	76	F	CP/wife	C	N/A			0	N/A	4 km– 1/week, No time	GP
											Ex is important	

P: participant number, PwPSP: person living with PSP, CP: care partner, Y: years, F: female, M: male, R: residential care, C: community

AF: atrial fibrillation, AFO: ankle foot orthosis, CC: cognitive change, CVD: cardiovascular disease, D: diabetes, DV: double vision, G: glaucoma, HT: hypertension, HC: hypercholesteremia, OA: osteoarthritis, MD: macular degeneration, SA: sleep apnoea, THR: total hip replacement, TSR: total shoulder replacement

C: chiropractor, CA: cardiologist, DI: dietitian, E: endocrinologist, EP: exercise physiologist, GP: general practitioner, HCP: health care provider, N: neurologist, O: ophthalmologist, OT: occupational therapist, P: psychologist, PT: physiotherapist, SP: speech pathologist

An overall finding arising from analysis of the interview data was that people living with a diagnosis of PSP and their care partners perceived that a range of intrinsic and extrinsic factors influence engagement in exercise and structured physical activity. Four main themes were identified: (i) there are beliefs and preferences about exercise and physical activity that impact on engagement; (ii) difficulty coping with disease progression impacts physical activity levels; (iii) there are facilitators to exercise participation in PSP; and (iv) there are perceived physical, psychological and economic barriers to engagement. The themes and sub-themes are presented in the following text, supported by quotations linked to the participants by code and transcription line numbers.

### Theme 1: There are beliefs and preferences about exercise that influence engagement

#### a) Engagement in exercise and structured physical activity is influenced by life experience with exercise

Participants reported that the extent to which they engaged in exercise and physical activity. This was related to their life-long practices in varied exercise activities and environments. Their past history of exercise participation and their capacity for daily physical activity influenced views about participating in structured physical activities after being diagnosed with PSP. (Box 1).

**Box 1. Prior exercise experience influences participation**

*“It's (exercise) part of my daily life and it is valuable for a whole lot of reasons*. *It's valuable for one thing*, *for getting to the exercise*…*I like to be strong” P5*, *line 26*, *849*

*“He was good at everything he did*. *You were a swimmer*, *you were a footballer*, *you were a cricketer—when he was young*. *Then we got married*, *so it was football and then it was taekwondo” P6 lines 42*

*“And it's the fact that XX has always been fit and strong that he actually needs good quality exercise and now well supervised exercise by someone who really knows what they're doing” P6*, *920–922*

*“They need to keep the muscles working*, *to walk*, *to eat*, *to talk*, *to do everything*. *We all need to do exercises*. *Particularly someone that’s got a disease that is going to limit their movement” P7*, *line 357–338*

*“When I was younger*, *I did hockey*, *exercises*, *and archery” P8*, *line 809*

*“Just before XX was diagnosed*, *she had a gym membership*. *We both had gym memberships there anyway…it was part of life anyway*. *So*, *we already had that*. *I mean XX’s had a gym membership longer than 20 years” P9*, *line 833*

*“We bought the tandem bike …we’ve been trying to go at least once a week*, *on the tandem…*. *And I thought*, *if the disease progresses*, *and we can’t ride the tandem*, *I can get a tandem trike*.*” P9 178*, *185*, *455*

*“I think exercise—the stronger and fitter you are*, *the better the fight you put up” P11*, *line 102*

*“(I do weight training) Pieces of equipment…I don’t like it…it’s pretty boring” P11*, *line 131*

*“I don’t like it (exercise)… I loved it (ballroom dancing) but I don’t like it now*. *I can’t do it…but the music can get me going… I used to love dancing but I don’t like anything else” 13*, *line 238*, *245*, *297*, *344*

*“But she doesn’t enjoy it (exercise) and there’s not the motivation to continue the exercise” P14*, *line 284*

*“I played cricket and squash*, *football*. *And I played tennis until I was 50” P16*, *line 137*

#### b) Engagement in exercise and structured physical activity is influenced by exercise preferences

Participants reported different preferences about types of exercise, and this influenced their choices and participation after PSP diagnosis. Many reported the importance of enjoyment and flexibility (Box 2).

**Box 2**

*“You’ve got to pick an exercise you really like doing… mine’s walking… Walking seven days a week with the dog…*. *But if I stop*, *I have trouble getting going again” P3 line 42*, *74*

*“I’m trying to get him back (to the gym) because they have proper machinery with proper weights and punching bags which is good*, *I’ve heard*, *for doing the punching when you’ve got Parkinson’s*. *P4 lines 240–244*

*“I had him doing Pilates with our physiotherapist*, *but he didn't love that because it wasn't physical enough” P6 line 73*

### Theme 2: Difficulty coping with the disease progression impacts activity

#### a) changes in ability affect activity engagement

Participants diagnosed with PSP reported difficulties with loss of mobility, independence and social connectedness. These deficits were associated with rapid disease progression, movement disorders and non-motor symptoms such as cognitive impairment, sleep difficulties, anxiety and depression. The most challenging symptoms included: falls, reduced mobility, vision, speech and swallowing impairments. These symptoms negatively affected participation in activities of daily living, exercises and physical activities (Box 3).

**Box 3. Changes in ability affect activity engagement**

*XX gets very tired if he has too big a day*, *and he’s taking a nap of an afternoon now so it sort of limits you a bit too” P3*, *line 274*

*“He used to go to the gym there every Monday by himself*, *but we're trying to maybe not do that because we feel that he needs*, *well*, *the girls at Steps certainly think he should not be exercising unsupervised because of the danger of falls” P6*, *line 68–70*

*“Boredom is a problem for XX now because there's always been work and physical stuff and now he doesn't quite know what to do with himself” P6*, *line 1006–1007*

*“He didn’t like going out very much*. *That could have been because*, *as he said to me*, *he can’t join in conversations and things*, *so he wanted to isolate himself” P7*, *line 165*

*“You feel a bit inadequate when you find that that’s all you can do at the time…I am aware of ongoing deterioration … I run out of energy very quickly*. *So I might only do the exercise bike two or three times a week… But it’s not a smooth line downwards*, *it fluctuates” P8*, *line 510*, *542*

*“Her vision started to deteriorate so that was a problem in facing any sort of physical exercise” P14*, *line 265*

*“He’s really tuned out*. *He’s got it*, *he’s got two problems and he doesn’t want to know any further*. *He’d like to be normal again*, *that’s all he thinks” P16*, *line 123*

#### b) Care partner strain and relationship changes impact on ability to assist with activity engagement

Care partners articulated elements of carer strain, a sense of social isolation and feelings of guilt, burden and frustration. There was acknowledgment of a transition in roles within the family as the disease progressed. Changes in roles were most often reported by the spouse, partner, child or sibling of the care-recipient. Changing roles, especially later in life, were sometimes associated with emotional tensions, including conflict and feelings of guilt. Care partners and family members often felt frustrated due to a lack of education available for community members, general practitioners and allied health clinicians about PSP (Box 4).

**Box 4. Care partner strain and relationship changes**

*I'd love to be there every day*, *taking her out*, *just to get her out of that joint*, *but obviously*, *I've got my family*, *I've got to make money” P2 line 497–499*

*“It doesn't happen unless I make it happen*. *How do people cope who are not intelligent*, *well educated*, *or have a bit of money in the bank*? *What do they do*? *And I feel angry about that” P6*, *line 412–413*

*“I said everybody is interested*, *everybody is supportive*, *everybody is sympathetic*, *but I am here 24/7 managing on my own and sometimes it's not easy” P6*, *line596-597*

*“We lost a lot of friends*. *The true ones stayed*, *but a lot of them… I think it’s because they don’t understand the condition” P7*, *line 205*

*“I’m full time carer…I only finished work last year*. *I worked shift work so*, *we could fit it in the morning before I went to work*, *or in the afternoon*. *So*, *we tried to fit it in” P9*, *line 349*, *355–356*

*“it’s a lot of driving for me… We are going to join ParkinSong and we’re going to ParkinDance and I take XX to his personal trainer” P12*, *line 382*

*“transport takes an enormous amount of time*, *not just the physical transport*, *getting in and out of the car and the waiting and finding parking and all of that… It was something as a carer that I’d never considered being a problem” P14*, *line 501*, *505*

*“It’s never easy*, *it’s not easy for anybody*. *It’s not easy*. *I do my best and I've always said I can only do my best” P16*, *line 573*

### Theme 3: Facilitators to exercise participation in PSP

#### a) Access to facilities and community groups enabled engagement

Participants reported that local facilities, for example swimming pools, and social connectedness in their local community helped them to keep active. For people living at home, the availability of local facilities such as gymnasiums, swimming pools, dance halls, bike tracks, walking paths and sports facilities was an important factor. Routines in daily living such as dog walking or participation in local groups such as bowls, dancing or bicycle riding were important. For those who lived in residential care, the availability of an in-house space for exercise classes, equipment and staffing support were enabling. Transport to and from the exercise room and supportive, actively involved staff, partners or other caregivers were key (Box 5).

**Box 5. Participation is facilitated by community engagement and activities**

*“I had the hobby (making model aeroplanes) since I was about 10*. *Brings great comfort to me …We belong to a club out there*, *and they fly there on a Saturday…(I like) the interaction with people*. *Talking about model aircraft and other things” P33 lines 65*, *108*, *114*

*“We have an exercise group here (at the Village)*, *or we have a couple*, *but the one I go to is here*, *once a week*, *and that’s gentle exercise… I sit on my wheelie*, *mainly because I now can’t push myself on a chair from a table or anything like that*. *And she has varied exercises*, *and we exercise with a partner*, *and we move from one set of exercises to another*.*” P8 line 462*, *469*

*“I go to the gym there (in the community)*. *The best part about it is the cup of coffee with our friends afterwards*. *There are days when I really don’t feel well enough to go*, *and I don’t feel like going*, *but because I have this carer come and take me*, *I go*. *A few of us have got to know each other*, *and we sit and have a cup of coffee afterwards together” P8*, *line 286–288*

*“There’s a lot of bike tracks here*. *We don’t have to put the bike on the car*. *We actually go from the front gate and we just ride” 93*, *line 390*

*“with ParkinSong*, *it’s a good thing to be singing*, *and people are happy singing*. *This is a good thing to do*, *and so is dance” P12*, *line 731*

*“That set us off to explore avenues of transport when I couldn’t do it so we’ve looked into that… Eastern Volunteers*, *we used them a couple of times when I was still—I could drop XX off to XX or XX but I couldn’t pick her up because I was doing something else so they did it” P14*, *line 476*, *481–482*

*“The social inclusion program at XX*, *a bus comes and picks her up*, *takes her back again… There’s a whole lot of things available*. *It’s getting to know about them and how to use them and coordinate them” P14*, *line 527*, *539*

#### b) Expert advice from health professionals was valued

Participants diagnosed with PSP, and the significant others in their lives, frequently sought guidance about safe exercises and general activities. Guidance was frequently sought from clinicians with experience working with people diagnosed with PSP and clinicians working in movement disorders clinics. Access to advice and therapy was frequently reported to be difficult, yet once attained, the services helped to improve understanding about PSP, mobility and falls prevention. People frequently sought this expert advice because they were unsure whether exercise would aggravate their PSP symptoms (Box 6).

**Box 6. Advice from health professionals is valued**

*“I made up a ramp*. *The physio said—helped me design and make up a ramp*. *Walk up the ramp and catch the ball” P3 line 488*

*“The GP’s always encouraging health*, *she’s a very healthy person herself*, *and she knows he walks the dog” P4*, *line 504*

*“XX is the exercise physiologist at XX*. *She's new*. *He's been through quite a few different ones*, *but this XX seems to be quite on the ball because she has contacted (the gym) about him being unsupervised” P6*, *line 855*

*“The physios have worked out—and the exercise physiologists*, *which I think they are—have worked out what*, *if you say*, *‘Look*, *I’m having trouble doing this*.*’ And they will suggest a range of exercises for you to do to help” P8*, *line 302–304*

*“You treat it (exercise) like medication*. *Oh*, *XX might do three sets of ten reps of a dumbbell*, *only one or two kilos…Physiotherapist said treat exercise like medication*. *We try and do it before each meal*. *Even a 15-minute session and XX’s neck straightens up” 93*, *line 148*, *154*

*“I think the physiotherapy has been good*. *With a special neurological physiotherapist*, *you’re finding out*, *doing these exercises” P9*, *line 517*

*“Well most of the instructions come from the physiotherapist*. *We—when XX was first diagnosed*, *we had some sessions at the Rehab*. *And we had a physio*, *speech*, *OT*. *So*, *we had a program for a while there and they gave us a whole lot of things” P9*, *line 600*

*“my personal trainer is a friend of mine*, *and he specialises in rehabilitation for people with strokes or Parkinson’s or MS…I go once a week” P11*, *line 96*

*“if they had specific PSP exercises that they told me that they would help me*, *I would do them” P13*, *line 304–305*

*“One of the things we found useful in getting advice from physios is when she falls how to get up and how to get her up” P14*, *line 841*

*“But she (the physio) was terrific*. *And she said*, *“There is really no point in giving him a huge amount of exercises*.*” She was interested in helping him around the house*, *what he might need*, *and she recommended he goes outside with a walker*, *which I've just purchased actually*, *the wheelie walker*, *because it just gives him confidence…she felt that just his mental condition wasn't going to be able to cope with it*. *It's true*, *because he tunes out” P16*, *line 162*, *181*

### Theme 4: There are perceived physical, psychosocial and economic barriers to accessing therapeutic exercise

#### a) disease progression and beliefs about limited treatment options are barriers to exercise engagement

Challenges to exercise and structured physical activity participation included the perceived rapid and fluctuating disease trajectory, physical and mental fatigue, behaviour changes and movement disorders. Co-morbid health conditions such as hypertension, visual disturbance and osteoarthritis were also reported to influence perceived exercise capacity (Box 7).

**Box 7. Fatigue is a barrier to exercise**

*“So XX gets tired if he has too big a day*, *and he’s taking a nap of an afternoon now so it sort of limits you a bit too” P4*, *line 274*

*“He gets mentally tired*, *I think*, *rather than physically tired*. *XX has got huge endurance*. *He can walk down to XX and back*, *although he's a bit wobbly by the time he gets back”*. *P6*, *lines 285*

*I’m pretty limited as to what I can manage… I can do it in small chunks*, *depending on what I can fit in and what I feel capable of on the day… it’s not go*, *go*, *go*, *go sort of exercise*. *I can’t do that anymore…*. *I often have to stop because I’m very tired” P8*, *491*, *755*

*“I have to be fairly picky*, *because I run out of energy very quickly*. *So*, *I might only do the exercise bike two or three times a week” P8*, *line 542*

*“Not a huge amount (of energy) …I’m very slow in my movements…my brain is slower” P11*, *line 457*

*“I’ve observed is that XX seems to have reasonable strength but what’s been changing is her abilities to use her strength in the right way*. *For example*, *getting in and out of the car*, *she doesn’t seem to move the legs and the body in sync that used to move*, *and she can’t” P14*, *line 345–347*

#### b) Falls risk and fear of falling limited activity

The need for supervision and environmental modifications was accelerated in the setting of a history of falls, reported fear of falling and limited independent engagement in activity. Barriers such as falls risk and reduced balance, strength and fitness may be overcome by exercise (Box 8).

**Box 8. Falls are a barrier to exercise**

*“He was mowing the lawn and I said*, *“Perhaps you should have a rest now”*. *No*, *he carried on*, *and that’s when he fell over*, *so he got—fell over twice” P4 lines368*

*“If he's getting out of a chair*, *he'll falls backwards*. *Normally I stand beside him*, *but at golf last week they had a coffee*, *I presume*, *and he fell right to the ground getting out of the chair*. *He's fallen in the kitchen leaning over*. *That's the other time he falls” P6*, *line 315*, *317–318*

*“I think XX is almost at the point of needing a hospital bed because we have a low king size bed*, *but he has struggles getting into it now that he falls into it and he's got the bar at the side which we organised*, *along with all the other things*, *to help him get up” P6*, *line 1025–1026*

*“It got to the stage he was falling too often and didn’t end up doing that (walking*, *bowls)” P7*, *line 264*

*“I wouldn’t trust him alone because of the safety aspects*. *Even if he was doing it here and fell*, *could have hit something” P7*, *line 440*

*“I think mainly when XX is down doing something which he shouldn’t do*, *which I think is like sweeping the leaves from the ground*. *And he physically has to do it—which I don’t think so*. *So that’s when he falls*, *especially when he’s getting up—he loses his balance”P12*, *line 579*

*“(I do fall but) Not very often because I’m so careful*. *I had a fall in respite… I fall backwards” P13*, *line 800*, *806*

*“XX doesn’t have frequent falls but she frequently feeling like she will fall” P14*, *line 860*

#### c) Access to facilities is limited by cost, financial resources and transport

All of the participants talked about their financial position and concerns about the costs of care, including exercise therapies. Movement disorders clinics had long wait times and private care incurred out of pocket expenses. Some people living with PSP became unable to drive or unsafe to use public transport alone and relied on others to access therapies (Box 9).

**Box 9. Financial resources and access to transport were barriers to exercise participation**

**Cost**

*“I think Mum's getting a bit more careful with money*, *because she realises that it's not a bottomless pit and it's not cheap*, *being in the Manor… she is funding herself” P2*, *line 790–791*

*“The tai chi’s not expensive*, *and it’s close by*. *The dancing is close by and free*, *it’s free*. *Ukulele is most expensive” P4*, *line 266*

*“We're not hard up*, *but because we are self- funded retirees*, *everything costs*. *We're not on a pension*. *It's quite different*. *But one time last year*, *I felt like I was just chucking dollar bills out the window really” 67*, *line 217*

*“Well*, *it’s money that you could be spending on something else*. *So that’s the barrier*. *But we’ve chosen to—I’ve chosen to look after myself*. *So*, *we do have private health insurance*, *which does help*, *but you only get a percentage back anyway” P11*, *line 292*

*“We’ve looked at taxi and getting taxi vouchers and that sort of thing…It’d cost us more money because we’re not eligible for the pension but that wouldn’t be a problem*. *If it was an emergency we just do it” P14*, *line 495*, *498*

**Transport**

*“I still drive a car*, *but I'm on the lowest legal level because I have a vision impairment*. *So*, *I'm on borrowed time (to drive him around)” P6*, *line 195*

*“He had a couple of bingles in the car because he couldn’t see the peripheral side of it…so he gave up driving” P7*, *line 98*

*“I book a carer to take me… They’ve got different carers*. *I have to work it out three or four weeks ahead at least” P8*, *line 672*

*“A few years ago*, *I was trying taxis and things*, *and that doesn’t work anymore… I had a couple of taxi drivers who wouldn’t load the wheelie into the vehicle*. *And they wouldn’t help me in and out of the car” P8*, *line 713*

#### d) Lack of credible information and limited access to experts

Participants wanted reliable and credible information about the range of available therapies, such as educational materials and links to support groups and organisation websites. All participants reported this to be difficult to find. Many participants experienced a difficult pathway to finding experienced and expert clinicians and movement disorders clinics (Box 10).

**Box 10. Credible information and expert clinicians**

*“I wanted to be more informed about why what I had I thought originally was just arthritis*, *was getting so much worse and beyond my control” P8*, *line 62–63*

*“I haven’t really known where to go for that sort of information (about exercise and treatment)…So I have never known where to go for information because I’ve never known what questions to ask*, *because I’ve never known what’s really been the matter” P8*, *line 1040*, *1044*

*“You’ve got to be going to reputable sites and know what you’re looking at*. *There’s a lot of rubbish on the net*. *But it’s like looking at clinical trials*, *you’ve got to go to the official site out what’s going on*. *And then from there*, *you’ll get links and you’ve got to look at actual medical papers written with authority” P9*, *line 84–85*

*“it was difficult—there’s not much information and people don’t tell you” P10*, *line 70*

*“No (information)*. *I was more or less told to look it up on the Internet” P11*, *line 27*

*“it is really quite confusing for people to understand the whole operation of the medical system*. *I had no concept of what an allied health professional was for example” P14*, *line 167–168*

## Discussion

Our investigations reflect the difficulty in diagnosing PSP, with often lengthy delays in patients receiving treatments that target the presenting symptoms [18]. People living with PSP reported seldom being referred for exercise therapy by their medical specialist. They reported navigating their own way to exercise by trial and error or by previous life experience of exercise. The participants also reported they did not know which forms of exercise and physical activity were helpful for people with PSP, echoing the findings of a recent systematic review [12]. Where there was a past history of exercise people understood the potential value of exercise. Where there was not a past history of exercise people were unclear about type and dosage.

Participants diagnosed with PSP and the care partners we interviewed were generally positive about exercising, and also reported several facilitators and barriers to participation in physical activities. The facilitators included: (i) being provided with information about the benefits of exercise and movement rehabilitation for PSP early on in the disease process or before provisional diagnosis but at a time when treatable symptoms are already manifest; (ii) having access to accurate information about PSP and its progression; (iii) access to healthcare providers, including medical, nursing and allied health, supportive of the role of exercise in PSP; (iv) having prior exercise experience and (v) having easy access to exercise facilities, groups and programs. Some of the barriers included: (i) perceptions of long delays in reaching a PSP diagnosis; (ii) lack of information about the benefits and contraindications to exercise and physical activity in PSP; (iii) fear of falling during physical activity; (iv) financial and transport constraints in getting to an exercise venue and (v) limited access to movement disorders specialists and PSP experts. These findings concur with the limited available published literature in atypical Parkinsonism [12, 13, 40] and the more extensive body of work in PD [22, 23, 50, 51].

All of the people with PSP that we interviewed experienced long delays in reaching a provisional diagnosis and often consulted multiple specialists. Moore et al (2014) reported similar findings and noted that a common misconception is that no treatment is available for PSP [52]. The participants also highlighted the need to develop a concise overview of symptoms and care options. Our respondents reported a lack of advice regarding management options and delays in referral to other healthcare providers, such as physiotherapists, podiatrists, occupational therapists and community-based exercise trainers. Arguably, the provision of early accurate information and referrals to movement disorders healthcare providers, who have experience with PSP, might enable the provision of comprehensive care in the period of diagnostic uncertainty and before major cognitive and physical decline. This approach is well established for PD [50, 53] and may also be suitable for PSP.

Care partner burden was a recurring theme in our sample of participants and is in agreement with recent international research in PSP and PD [54, 55–57]. The care partners were often older and reported high levels of strain and fatigue associated with their carer roles and the time spent seeking out resources, services and providing transportation. Caregiver burden is a recognised phenomenon with families and care partners finding themselves educating healthcare providers, advocating for the needs of people with PSP and attempting to navigating complex health and social care systems [52].

A history of exercise participation and an interest in structured physical activities facilitated exercise engagement. Participants in the present study believed that provision of exercise and activity facilities within the local community was important. For example, sport and activity participation in local facilities such as parks, bicycle paths, walking tracks and gyms [58, 59]. Community engagement and connection to others through hobbies, social groups and peer-support was viewed as important. These have been demonstrated to influence and facilitate well-being in other neurological conditions [37, 52, 60].

All of the participants reported that fear of falling, consequences of falls and physical limitations influenced their beliefs about the safety of structured physical activities. All reported an increased incidence of falls and near-misses in the period when they were awaiting a definitive diagnosis. These self-reports may be useful for obtaining an earlier diagnosis of PSP [18]. Furthermore, limiting participation in the presence of communication difficulties and cognitive impairment were reported to hamper interactions with others and to increase social isolation. Similar findings were noted by Moore (2014) and Wiblin (2017) [52, 60].

Results of this study need to be interpreted with caution in light of several limitations. Although we reached saturation of data for key themes, the results reflected the views of 16 people and might not represent all people living with PSP. Diagnostic uncertainty and the heterogeneity of the disease itself also could have limited generalisability. Specificity of findings to the people involved and their context with respect to access to community services, health systems, and financial constraints might also limit generalisability. Bias of the researchers may have influenced interpretation, although measures were taken to control bias in the research design.

To conclude, people with PSP and their care-partners were receptive to the value of therapeutic exercises and structured physical activities. Care recipients and healthcare professionals generally did understand that exercise is important for strength, endurance and participation in ADL. Not all of them had access to specific guidance about the optimal type and dosage of exercises and physical activities for people living with atypical Parkinsonism. There was no prior evidence on the potential disease-specific benefits of exercise for people with PSP. After being diagnosed with PSP there was often a prolonged period of inactivity at a time when exercise appears to have the greatest potential to mitigate disability.

### Highlights

- People with PSP and their care partners seek early and reliable information about the benefits, and precautions for, different types of exercise and physical activity
- Individual exercise preferences and capabilities need to be considered when designing therapeutic exercise programs for people living with PSP
- Access to home, healthcare and community facilities for safe exercises and physical activity is needed
- User-friendly evidence-based information is needed on the potential benefits of exercise and structured physical activities for movement disorders, falls prevention and social participation

Clinical guidelines are needed on targeted exercises, education and structured physical activities for people living with PSP

## Supporting information

S1 Checklist Consolidated criteria for reporting qualitative studies (COREQ): 32-item checklist. (DOCX)
